# Telomere Length and Arterial Stiffness Reflected by Brachial–Ankle Pulse Wave Velocity: A Population-Based Cross-Sectional Study

**DOI:** 10.3390/jpm11121278

**Published:** 2021-12-02

**Authors:** Kyi Mar Wai, Sawada Kaori, Ken Itoh, Okuyama Shinya, Yuka Uchikawa, Sakura Hayashi, Akiko Shiraki, Koichi Murashita, Shigeyuki Nakaji, Kazushige Ihara

**Affiliations:** 1 Department of Social Medicine, Graduate School of Medicine, Hirosaki University, Hirosaki 036-8562, Japan; iwane@hirosaki-u.ac.jp (S.K.); s.okuyama74@gmail.com (O.S.); nakaji@hirosaki-u.ac.jp (S.N.); ihara@hirosaki-u.ac.jp (K.I.); 2 Department of Human Ecology, Graduate School of Medicine, The University of Tokyo, Tokyo 113-0033, Japan; 3 Center of Advanced Medical Science, Department of Stress Response Science, Graduate School of Medicine, Hirosaki University, Hirosaki 036-8562, Japan; itohk@hirosaki-u.ac.jp; 4 Department of Mibyo Science, Graduate School of Medicine, Hirosaki University, Hirosaki 036-8562, Japan; 5 Research and Development Division, MiRTeL Company Limited, Hiroshima 734-0001, Japan; yuka_uchikawa@mirtel.co.jp; 6 Business Development Division, MiRTeL Company Limited, Hiroshima 734-0001, Japan; hayashi@mirtel.co.jp; 7 Inspection Division, MiRTeL Company Limited, Hiroshima 734-0001, Japan; shiraki@mirtel.co.jp; 8 Center of Innovation, Research Initiatives Organization, Hirosaki University, Hirosaki 036-8562, Japan; murasita@hirosaki-u.ac.jp

**Keywords:** telomere, G-tail length, pulse wave velocity, arterial stiffness, Japan

## Abstract

Telomere (TL) is a biomarker of biological aging, and its shortening is associated with major risk factors for cardiovascular diseases (CVD). This study aimed to identify whether TL is associated with arterial stiffness as reflected by brachial–ankle pulse wave velocity (baPWV). This population-based cross-sectional study involved 1065 individuals in the Iwaki area, Japan. Total TL length and TL G-tail length were measured by hybridization protection assay. The baPWV was measured on the right and left sides using a non-invasive vascular screening device. The associations between TL and baPWV were assessed by multivariate linear regression. Compared with the shortest total TL tertile, the longest total TL group showed a significant decrease in baPWV (lowest vs. highest tertile: adjusted beta: −41.24, 95% confidence interval (CI): −76.81, −5.68). The mean baPWV decreased with a longer TL (TL G-tail length: *p* trend < 0.001, total TL: *p* trend < 0.001). TL G-tail and total TL lengths were inversely associated with baPWV, implicating TL shortening in the development of CVD. This study provides evidence of the factors influencing CVD risks at a very early stage when individuals can still take necessary precautions before CVD gives rise to a symptomatic health outcome.

## 1. Introduction

Telomeres (TLs) are repeated sequences of TTAGGG oligomer caps at the chromosomal ends [1,2]. TLs are involved in maintaining cellular integrity by protecting end-to-end chromosomal fusion [1,3]. TLs shorten with each cell division, and subsequent cellular senescence occurs when they reach a critical length [1]. DNA polymerase fails to fully replicate at the terminal 3′-end of DNA, resulting in the extension of G-rich repeats known as the TL G-tail [4]. The G-tail plays a critical role in the formation of the T-loop, essential for hiding the terminal end of TLs and preventing end-to-end fusion [2,5]. In addition to total TL length, telomere G-tail length also acts as a marker of biological aging.

Premature biological aging may contribute to the development of age-related diseases. Since chronological aging is a critical factor of cardiovascular diseases (CVD), there is overwhelming interest around whether a biological aging biomarker, TL, could predict the risk of developing CVD [6]. In particular, TL shortening is associated with age-related pathophysiological outcomes such as CVD and malignancy [7,8]. TL shortening is reportedly associated with hypertension, atherosclerosis, and vascular dementia [8,9,10,11]. Increased oxidative stress and inflammation may accelerate TL shortening and endothelial cell turnover [12]. 

In developed countries, CVD remains a major cause of death with high prevalence and consequences of disease burden [13,14]. Arterial stiffness is a well-recognized contributor to the pathogenesis of CVD [15]. Pulse wave velocity (PWV) reflects the stiffness of muscular and elastic arteries and is an independent risk factor for CVDs [15]. Brachial–ankle PWV (baPWV) is non-invasive, simple to measure, and is frequently used to assess CVDs and/or their risk factors [15,16]. Meanwhile, a shortened TL was previously reported to be associated with an increased PWV due to impaired hemodynamic stress in the arterial intima or media [17,18,19].

Given the global burden of CVD, it is important to examine whether TL shortening is a proxy for the early identification of developing CVD and its risks. Although there are many studies regarding the associations between TL and CVD, few epidemiological studies have focused on the possible associations of TL and non-invasive assessment of arterial stiffness reflected by PWV. Moreover, G-tail length, rather than the total TL length, serves as a triggering point for cellular viability and senescence [5]. A possible association between telomere G-tail length and arterial stiffness has not been investigated, although a shorter TL reportedly triggers cellular senescence [5,20]. Therefore, this study aims to determine whether total TL and TL G-tail length are associated with arterial stiffness reflected by baPWV in the general Japanese population. 

## 2. Materials and Methods

### 2.1. Study Design and Population

This cross-sectional design used data originating from the Iwaki Health Promotion Project (IHPP) in 2019. The rationale, design, and perspectives of the IHPP have been described elsewhere [21]. In brief, the IHPP is an annual health checkup among community dwellers more than 19 years old, residing in the Iwaki area, Aomori Prefecture, Japan. Aomori Prefecture is reported as the prefecture with the shortest life expectancy in Japan. The mortality rate of Aomori Prefecture was 1.3–1.9-fold higher than that of Nagano Prefecture, which has the highest life expectancy in Japan among >20-year-old adults [21]. The IHPP examines the lifestyle and general health status of the community using questionnaires and biological parameters. In 2019, a total of 1065 individuals participated in the IHPP. The Research Ethics Committee of Hirosaki University approved the study protocol (No.2019-009), and all participants provided written informed consent before enrollment.

### 2.2. Body Anthropometry and Biochemical Measurements

The participants were interviewed by trained interviewers to assess their medical and personal history such as age and smoking and drinking statuses. Height and weight were assessed, and body mass index (BMI) was calculated as body weight (kg) ÷ height (m^2^). Fasting venous blood samples were taken from the antecubital vein and stored as serum or whole blood at −80 °C for subsequent analysis. Blood sugar, HbA1c, low-density lipoprotein (LDL), high-density lipoprotein (HDL), triglycerides (TG), and C-reactive protein (CRP) levels were determined by an enzyme assay method in an accredited laboratory (LSI Medience Corporation, Tokyo, Japan).

### 2.3. Brachial–Ankle PWV Measurement 

In this study, arterial stiffness was reflected by baPWV, measured by trained staff using a non-invasive vascular screening device (BP-203RPE III, Omron Colin Co., Ltd., Tokyo, Japan). This volume–plethysmography apparatus simultaneously records PWV, blood pressure, electrocardiogram, and heart sounds. The detailed procedure and theory are explained elsewhere [16]. In brief, participants were examined in the supine position with cuffs wrapped around both the brachia and ankles and the electrocardiogram electrodes placed on both wrists. The cuffs were connected to sensors that determine BP or volume pulse form. The baPWV was obtained using the formula baPWV = (La − Lb) ÷ ∆Tba, where La is the superficial measurement estimated from the path length from the suprasternal notch to the ankle, using the equation La = (0.8129 × height of the patient (in cm) + 12.328); Lb is estimated from the path length from the suprasternal notch to the brachium using the equation Lb = 0.2195 × height of the patient (in cm) − 2.0734; and ∆Tba is the time interval between the wavefront of the brachial waveform to that of the ankle waveform. According to the Physiological Diagnosis Criteria for Vascular Failure Committee, a baPWV value of 1400 cm/s is reported to be useful to screen for atherosclerosis among the general population (normal: baPWV < 1400 cm/s; borderline: baPWV 1400–1800 cm/s; vascular smooth muscle dysfunction; baPWV > 1800 cm/s) [22]. The intra- and inter-observer reproducibility coefficients have been confirmed previously as 8.4% and 10.0%, respectively [15]. In this study, the baPWV was measured on the right and left sides, and the mean was calculated. 

### 2.4. Telomere Length Measurement 

DNA was extracted from whole blood samples by the modified phenol–chloroform method within 24 h. Total TL length and TL G-tail length were measured by hybridization protection assay (HPA) as described previously [23]. HPA is suitable for large-scale assessment using a 96-well format luminometer. In brief, the telomere HPA probe was added to 5 μg of nondenatured genomic DNA and incubated at 60 °C for 20 min. Next, a hybridization buffer was added to each sample and again incubated at 60 °C for 10 min. The plates were subjected to luminescence measurement using a luminometer. The sensitivity of TL G-tails measured by the HPA method was assessed using synthetic telomeric DNA constructs of 10–62-nucleotide G-tail length. The coefficients of variation were less than 10% for TL G-tail length and total TL length.

### 2.5. Statistical Analysis

All statistical analyses were performed using STATA 13.1 (StataCorp LP, College Station, TX, USA). Data were expressed as means, standard deviations (SD), or percentages. The mean differences across the tertiles of TL G-tail length and total TL length were determined using a one-way analysis of variance test for continuous variables and chi-squared test for categorical variables. Since CRP and TG were highly left-skewed, the Kruskal–Wallis test was applied. Mean baPWV was considered an outcome variable and was calculated by the average of the right and left baPWV values. Pearson’s correlation coefficient was used to assess the correlations between the right side, left side, and mean baPWV values. In this study, the associations between TL and arterial stiffness as reflected by PWV were determined by multivariate linear regression adjusting for confounders. The study included confounders of age, sex, smoking status, alcohol drinking, hypertension, blood sugar, C-reactive proteins, BMI, and lipid profile to cover the role of lifestyle and pre-existing conditions based on existing studies [17,24,25,26,27]. Values of *p* < 0.05 were considered indicative of significance in all analyses. 

## 3. Results

**Figure 1:**
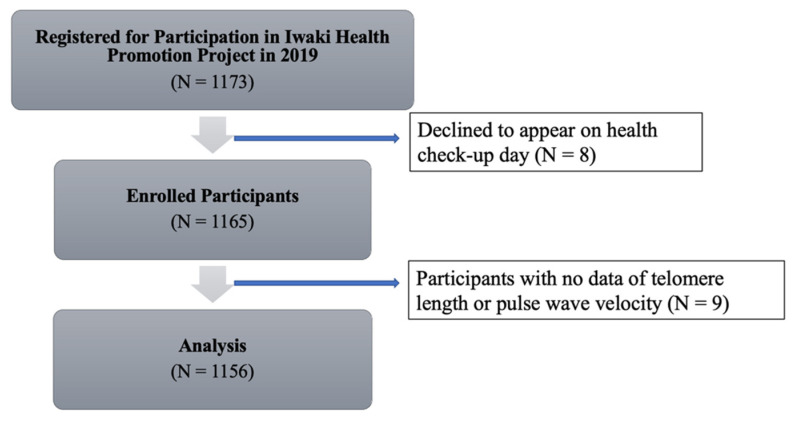
A participants’ flow diagram.

A total of 1073 individuals registered to participate in IHPP in 2019. Of these, eight participants declined to attend on the health check-up day. As shown in Figure 1, this study analyzed the data of 1056 participants. The characteristics of the study participants are listed in Table 1. The mean age of the participants was 52 years (standard deviation (SD): 15.2). About 18% of the participants had hypertension, defined as systolic blood pressure ≥140 mmHg and/or diastolic blood pressure ≤90 mmHg. TL was assessed as both TL G-tail length and total TL, and the mean values were calculated (mean TL G-tail length: 31,664.2 RLU/µg DNA; mean total TL: 356,478.1 RLU/µg DNA). The mean baPWV of the participants was 1415.2 cm/s (SD: 340.5 cm/s), and the right, left, and mean baPWV were strongly correlated (Table 2: r > 0.98, *p* < 0.001).

Table 3 shows significant differences between characteristics across the tertiles of TL G-tail length and total TL length. In the longest TL group, the mean age was significantly younger compared with the shortest TL group (TL G-tail: 48.3 vs. 56.9 years, *p* < 0.001; total TL: 45.5 vs. 59.7 years, *p* < 0.001). Similarly, baPWV was significantly lower with a longer TL (TL G-tail: 1361.1 vs. 1469.6 cm/s, *p* < 0.001; total TL: 1299.2 cm/s vs. 1552.9 cm/s, *p* < 0.001). Across TL G-tail tertiles, there were significant differences in BMI (longest TL vs. shortest TL: 22.4 vs. 23.5 kg/m^2^, *p* < 0.001), triglycerides (longest TL vs. shortest TL: 88.0 vs. 108.8 mg/dL, *p* = 0.004), and blood sugar (longest TL vs. shortest TL, 92.4 vs. 100.4 mg/dL, *p* < 0.001). 

The results of multivariate linear regressions of TL and baPWV are summarized in Table 4. After adjustment for potential confounders, the longest total TL showed a significant decrease in baPWV compared with the shortest total TL tertile (lowest tertile vs. highest tertile: adjusted beta: −41.24, 95% confidence interval (CI): −76.81, −5.68). Other significant explanatory variables for an increased baPWV were age ≥60 years (adjusted beta: 441.47, 95% CI: 403.74, 479.20), hypertension (adjusted beta: 195.62, 95% CI: 159.30, 231.93), high TG (adjusted beta: 0.17, 95% CI: 0.01, 0.34), high blood sugar (adjusted beta: 3.40, 95% CI: 2.47, 4.34), and high C-reactive protein (adjusted beta: 57.62, 95% CI: 28.87, 86.36). In contrast, baPWV was negatively associated with being female (adjusted beta: −92.27, 95% CI: −127.13.74, −60.40) and BMI (adjusted beta: −4.67, 95% CI: −8.81, −0.54). No significant association was observed between TL G-tail length and baPWV; however, the other explanatory variables showed similar findings to those of total TL in the adjusted model of TL G-tail length and baPWV. 

**Figure 2:**
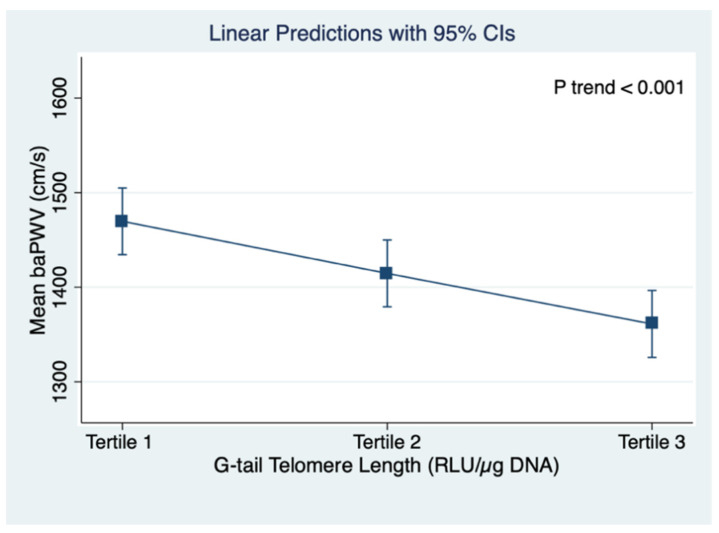
Linear regression predictions of mean baPWV and G-tail telomere length across the tertiles of G-tail telomere length; baPWV: brachial–ankle pulse wave velocity; CI: confidence intervals. Tertile 1: 11,854.32~29,611.05 RLU/µg DNA, Tertile 2: 29,611.05~33,409.38 05 RLU/µg DNA, and Tertile 3: 33,409.38~53,575.0605 RLU/µg DNA).

**Figure 3:**
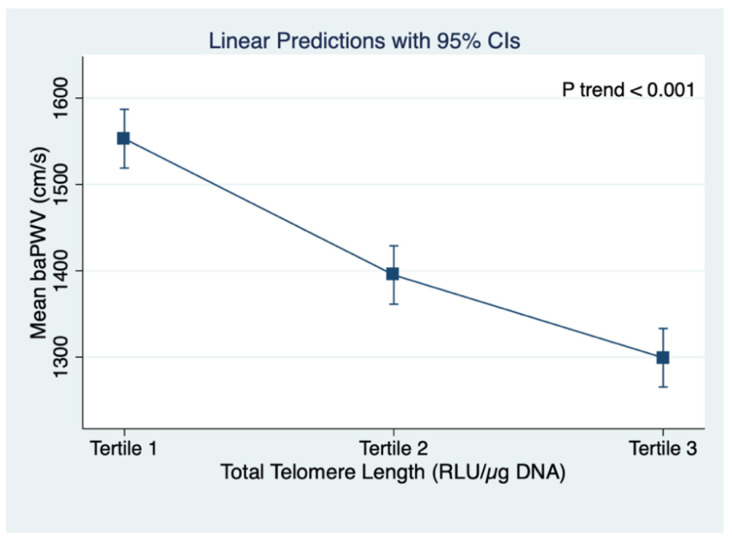
Linear regression predictions of mean baPWV and total telomere length across the tertiles of total telomere length, baPWV: brachial–ankle pulse wave velocity; CI: confidence intervals. Tertile 1: 103,771.35~334,193.49 RLU/µg DNA, Tertile 2: 334,193.49~376,734.07 RLU/µg DNA, and Tertile 3: 376,734.07~596,420.43 RLU/µg DNA).

Figure 2 shows the linear regression predictions of mean baPWV and TL G-tail length across the tertiles of TL G-tail length. The mean baPWV decreased with longer TL G-tail length (*p* trend < 0.001). Similarly, a significant negative trend was found between mean baPWV and total TL (*p* trend < 0.001), as shown in Figure 3.

## 4. Discussion

This is the first report of associations of TL G-tail length and total TL length with arterial stiffness as reflected by baPWV in a general population. There was a highly significant association between TL shortening and increased baPWV, a CVD risk factor. Therefore, TL may be associated with vascular function across the course of an individual’s life and may act as an underlying factor in the pathogenesis of CVD. 

The main finding of this study was a negative association between TL and baPWV, providing insight into the link between biological aging and vascular aging. Total TL length was significantly negatively associated with baPWV after adjusting for confounders. Although there was no significant association between G-tail TL and baPWV in the adjusted models, we identified a clear decreasing trend of baPWV with a longer total TL or G-tail TL (Table 3). The G-tail structures might be more sensitive to the magnitude of individual differences in cellular variability than total TLs [28,29,30]. Nevertheless, the results are in line with prior reports that a shorter TL was associated with arterial stiffness [17,19,24,25,26]. In a cross-sectional study, men with a shorter TL showed increased PWV, suggesting that TL could serve as an indicator of the age-dependent increase in arterial stiffness [19]. A larger community-based study in South Africa also reported an inverse association between TL and PWV with no interaction with gender or menopausal status [24]. Moreover, a previous longitudinal study showed that accelerated biological aging was correlated with early atherosclerosis by showing a positive association between TL attrition and carotid intima–media thickness [26]. Hence, together with prior results, this study supports the notion that TL attrition independently contributes to CVD outcomes by increasing arterial stiffness as reflected by PWV.

The current study identified a decreasing trend of baPWV with longer total TL or TL G-tail length. Leucocyte TL may shorten in parallel with that of endothelial cells [31]. Leucocyte TL shortening is secondary to chronic inflammation rather than the aging of endothelial cells [31]. It is possible that chronic inflammation accelerates cellular TL turnover, ultimately leading to the development of atherosclerosis. On the other hand, hemopoietic stem cells, including endothelial progenitor cells, are involved in the pathological repair of endothelial tissues [32,33]. Thus, immune effector cells and hemopoietic stem cells might induce leucocyte TL shortening, resulting in altered endothelial repair and atherosclerosis. Another factor contributing to arterial stiffness is chronological aging. In the course of aging, arteries become stiffer due to alteration of the composition of elastin [34]. Elastin undergoes fragmentation and degradation, being replaced by the much stiffer collagen. As a result, resting vascular smooth muscle tone is tensed and arterial stiffness increased [34]. Since TL is a well-recognized biomarker of biological aging, our findings suggest that biological aging accompanies chronological aging in a parallel fashion with regard to arterial stiffness.

In the present study, arterial stiffness was evaluated using baPWV, which is commonly used in practical and/or clinical settings among the Japanese population [15,16]. Measurement of PWV is recommended to evaluate subclinical organ damage because PWV is strongly associated with CVD incidents [35,36,37]. Although there has been debate about whether baPWV reflects aortic arterial stiffness, several studies have shown its validity and prognosis utility [16,35,36]. A recent large meta-analysis showed that baPWV independently predicts CVD outcomes [33]. Several cross-sectional and cohort studies demonstrated that baPWV was significantly positively associated with CVD events and CVD markers in both the general population and patients [37,38,39,40]. For example, among the general Japanese population, the adjusted hazard ratio of total CVD events was 2.7 in subjects of baPWV >18 m/s [35]. Hence, baPWV may be useful for assessing subclinical damage in population-based settings. The findings of this study will facilitate early screening for CVD risk in the community.

Considering that TL is associated with arterial stiffness across the life course, our findings offer an important insight into TL as an underlying factor for the development of CVD. Approximately 18.6 million adults died in 2019 from CVD worldwide, and CVDs stand as one of the most common causes of death [14]. Thus, it is important to understand the factors influencing CVD risks at a very early stage and take necessary preventive actions at the community level. Meanwhile, large-scale screening with high validity and reproducibility is valuable in a community setting. Considering the advantage of high-throughput and large-scale screening in a population-based setting, this epidemiological study provides evidence of the relationship between CVD risk and total TL and TL G-tail length in a general population.

The current study has several limitations. First, although carotid–femoral PWV is regarded as “the gold standard” for arterial stiffness assessment, this study measured baPWV to assess arterial stiffness, because baPWV measurement is more commonly used in Japan and has high validity and reproducibility [16]. Second, despite the fact that a couple of confounders of TL and baPWV were adjusted for, the role of genetic contribution cannot be clarified in this study. The study may also provide limited explanation for the role of other unadjusted confounders, such as lifestyle and pre-existing conditions. Moreover, possible effects of medication and drug history on arterial stiffness were not adjusted for in the analysis due to the inaccessibility of detailed information. Finally, the current study cannot identify the causal relationship nor mechanistic pathway between TL shortening and baPWV. Further investigations of a longitudinal design may help to figure out this unsolved hypothesis.

## 5. Conclusions

In conclusion, the present study identified that the shortening of TL G-tail length or total TL length were independent predictors of increased baPWV in a general Japanese population. The findings suggest that TL shortening may act as an underlying factor for the development of CVD. Assessment of TL would be useful for the early detection of arterial stiffness and/or CVD before it gives rise to a symptomatic health outcome. 

## Figures and Tables

**Table 1:** Basic characteristics of participants (*n* = 1065).

Characteristics	Mean ± SD	*n* (%)
**General Characteristics**		
Age (years)	52.7 ± 15.2	
Sex		
Male		435 (40.9)
Female		630 (59.1)
Smoking status		
Non-smokers		673 (63.8)
Current smokers		182 (17.2)
Past smokers		200 (19.0)
Alcohol drinking status		
Non-drinkers		504 (47.9)
Current drinkers		504 (48.0)
Past drinkers		43 (4.1)
Body mass index (kg/m^2^) ‡	23.0 ± 3.6	
Underweight		82 (7.7)
Normal		700 (65.7)
Overweight		230 (21.6)
Obese		53 (4.9)
Systolic blood pressure (mmHg)	120.8 ± 16.9	
Diastolic blood pressure (mmHg)	76.8 ± 11.3	
Diabetes, yes		43 (4.0)
Hypertension, yes		202 (18.9)
Right baPWV (cm/s)	1411.2 ± 343.3	
Left baPWV (cm/s)	1419.3 ± 341.0	
Mean PWV (cm/s)	1415.2 ± 340.5	
**Laboratory Findings**		
Blood sugar (mg/dL)	96.1 ± 16.3	
Triglyceride (mg/dL) †	77 (55–114)	
LDL Cholesterol (mg/dL)	116.1 ± 29.8	
HDL Cholesterol (mg/dL)	65.0 ± 16.6	
HbA1c (%)	76.8 ± 11.3	
C-reactive protein (mg/dL) †	0.03 (0.02–0.08)	
Telomere G-tail length (RLU/µg DNA)	31,664.2 ± 4744.3	
Total telomere length (RLU/µg DNA)	356,478.1 ± 50,678.3	

SD: standard deviation; baPWV: brachial–ankle pulse wave velocity; HDL: high-density lipoproteins; LDL: low-density lipoproteins. ‡ Body mass index is classified into four groups as underweight (<18.5 kg/m^2^), normal (18.5 and 18.5–25 kg/m^2^), overweight (25 and 25–30 kg/m^2^), and obese (>30 kg/m^2^). † Median and interquartile range values are expressed.

**Table 2:** Correlation matrix of right baPWV, left baPWV and mean PWV (Pearson’s correlation, *n* = 1056).

Variables	Right baPWV (cm/s)	Left baPWV (cm/s)	Mean baPWV (cm/s)
Right baPWV (cm/s)	1		
Left baPWV (cm/s)	0.981 *	1	
Mean baPWV (cm/s)	0.995 *	0.995 *	1

* *p* < 0.001; baPWV: brachial–ankle pulse wave velocity.

**Table 3:** Characteristics according to telomere G-tail length tertiles and total telomere length tertiles (*n* = 1056).

Variables	Telomere G Tail Length (RLU/µg DNA) ^a^			*p*-Value ^†^	Total Telomere Length (RLU/µg DNA) ^b^			*p*-Value ^†^
	Tertile 1 Mean ± SD	Tertile 2 Mean ± SD	Tertile 3 Mean ± SD		Tertile 1 Mean ± SD	Tertile 2 Mean ± SD	Tertile 3 Mean ± SD	
Age (years)	56.9 ± 14.9	52.7 ± 15.2	48.3 ± 14.5	<0.001	59.7 ± 13.7	52.8 ± 14.8	45.5 ± 13.8	<0.001
Sex, male ^‡^	140 (13.2)	144 (13.5)	151 (14.2)	0.676	168 (15.8)	138 (12.9)	129 (12.1)	0.008
Body mass index (kg/m^2^)	23.0 ± 3.5	23.1 ± 3.6	22.8 ± 3.7	0.587	23.5 ± 3.6	23.0 ± 3.6	22.4 ± 3.6	<0.001
Systolic blood pressure (mmHg)	122.5 ± 16.5	121.1 ± 17.1	118.8 ± 17.0	0.015	123.5 ± 17.0	121.9 ± 17.4	116.9 ± 15.7	<0.001
Diastolic blood pressure (mmHg)	77.2 ± 10.6	76.8 ± 10.7	76.6 ± 12.6	0.771	77.9 ± 10.9	77.0 ± 11.8	75.6 ± 11.2	0.021
Hypertension, yes ^‡^	74 (0.9)	66 (6.2)	62 (5.8)	0.514	81 (7.6)	74 (6.9)	47 (4.4)	0.003
Right baPWV (cm/s)	1467.6 ± 347.8	1408.8 ± 334.6	1356.8 ± 339.2	<0.001	1547.4 ± 380.6	1390.5 ± 308.2	1297.1 ± 287.7	<0.001
Left baPWV (cm/s)	1471.7 ± 344.0	1420.4 ± 335.4	1365.5 ± 336.2	<0.001	1558.5 ± 374.5	1399.7 ± 306.8	1301.3 ± 286.2	<0.001
Mean baPWV (cm/s)	1469.6 ± 343.9	1414.6 ± 333.3	1361.1 ± 336.3	<0.001	1552.9 ± 375.3	1395.1 ± 306.1	1299.2 ± 285.4	<0.001
Blood sugar (mg/dL)	96.7 ± 15.7	96.4 ± 16.6	95.0 ± 16.6	0.348	100.4 ± 20.1	95.3 ± 15.1	92.4 ± 11.4	<0.001
Triglyceride (mg/dL) ^§^	95.9 ± 99.1	103.6 ± 90.9	93.1 ± 58.5	0.233	108.8 ± 101.5	95.9 ± 78.1	88.0 ± 70.2	0.004
LDL Cholesterol (mg/dL)	116.1 ± 29.2	115.5 ± 29.5	116.8 ± 30.7	0.840	120.0 ± 28.6	116.3 ± 31.6	112.0 ± 28.6	0.002
HDL Cholesterol (mg/dL)	65.9 ± 16.6	64.1 ± 16.7	65.0 ± 16.5	0.357	63.7 ± 16.3	64.9 ± 16.1	66.4 ± 17.2	0.081
HbA1c (%)	5.8 ± 0.7	5.7 ± 0.5	5.7 ± 0.6	0.092	5.9 ± 0.7	5.7 ± 0.6	5.6 ± 0.5	<0.001
C-reactive protein (mg/dL) ^§^	0.1 ± 0.4	0.1 ± 0.3	0.2 ± 0.6	0.305	0.1 ± 0.4	0.1 ± 0.6	0.1 ± 0.3	0.411

SD: standard deviation; baPWV: brachial–ankle pulse wave velocity; HDL: high-density lipoproteins; LDL: low-density lipoproteins. ^a^ Tertile 1: 11,854.32~29,611.05 RLU/µg DNA, Tertile 2: 29,611.05~33,409.38 RLU/µg DNA, Tertile 3: 33,409.38~53,575.06 RLU/µg DNA. ^b^ Tertile 1: 103,771.35~334,193.49 RLU/µg DNA, Tertile 2: 334,193.49~376,734.07 RLU/µg DNA, Tertile 3: 376,734.07~596,420.43 RLU/µg DNA. ^†^ *p*-values were derived from a one-way ANOVA test for continuous variables and a chi-squared test for categorical variables. ^§^ *p*-values were derived from a Kruskal–Wallis test. ^‡^ Number and percentage values are expressed.

**Table 4:** Multivariate linear regression of telomere length and brachial–ankle pulse wave velocity (*n* = 1056).

Variables	TL G-Tail and Mean baPWV	Total TL and Mean baPWV
	Adjusted Beta (95% CI)	Adjusted Beta (95% CI)
Telomere G-tail length (RLU/µg DNA)		
Tertile 1 (11,854.32~29,611.05)	ref	
Tertile 2 (29,611.05~33,409.38)	−3.55 (−36.58, 29.48)	
Tertile 3 (33,409.38~53,575.06)	1.88 (−31.99. 35.75)	
Total telomere length (RLU/µg DNA)		
Tertile 1 (103,771.35~334,193.49)		ref
Tertile 2 (334,193.49~376,734.07)		−52.44 (−86.06, −18.83) **
Tertile 3 (376,734.07~596,420.43)		−41.24 (−76.81, −5.68) *
Age		
≤40 years	ref	ref
40–60 years	125.59 (90.43, 160.76) ***	119.32 (83.92, 154.71) ***
≥60 years	455.01 (418.36, 491.67) ***	441.47 (403.74, 479.20) ***
Sex (ref: male)	−93.58 (−125.58, −61.54) ***	−92.27 (−124.13, −60.40) ***
Smoking (ref: non-smokers vs. current smokers)	17.64 (−19.98, 55.27)	13.31 (−24.21, 50.83)
Alcohol drinking (ref: non-drinkers vs. current drinkers)	4.44 (−25.65, 34.54)	2.62 (−27.58, 32.84)
Hypertension (ref: no)	193.43 (156.97, 229.91) ***	195.62 (159.30, 231.93) ***
Triglyceride (mg/dL)	0.19 (0.02, 0.36) *	0.17 (0.01, 0.34) *
Blood sugar (mg/dL)	3.54 (2.59, 4.47) ***	3.40 (2.47, 4.34) ***
Body mass index (kg/m^2^)	−4.62 (−8.77, −0.47) *	−4.67 (−8.81, −0.54) *
C-reactive protein (mg/dL)	56.95 (28.03, 85.87) ***	57.62 (28.87, 86.36) ***

*p < 0.05; ** *p* < 0.01; *** *p* < 0.001. TL: telomere; baPWV: brachial–ankle pulse wave velocity. Adjusted for age, sex, smoking status, alcohol drinking status, hypertension, triglyceride, blood sugar, body mass index, and C-reactive protein.

## Data Availability

The data that support the findings of this study are available from the corresponding author (KMW), upon reasonable request.
